# Protection by glia-conditioned medium in a cell model of Huntington disease

**DOI:** 10.1371/4fbca54a2028b

**Published:** 2012-07-02

**Authors:** Carolina Ruiz, Maria Jose Casarejos, Ana Gomez, Rosa Solano, Justo Garcia de Yebenes, Maria Angeles Mena

## Abstract

The physiological role of huntingtin and the pathogenic mechanisms that produce the disease are unknown. Mutant huntingtin changes its normal localization and produces cytoplasmic and intranuclear inclusions, changes gene transcription, alters synaptic transmission, impairs mitochondrial activity and activates caspases and other pro-apoptotic molecules, promotes excitotoxicity, energy deficits, synthesis and release reduction of neurotrophic factors and oxidative stress. Previous studies confirm that the mutant huntingtin difficult neurotrophic function of astrocytes leading to neuronal dysfunction in Huntington’s disease. Our objective was to study the neuroprotective potential role of glia-conditioned medium (GCM) in an in vitro model of Huntington’s disease. We used conditionally-immortalized striatal neuronal progenitor cell lines (STHdhQ7/Q7 and STHdhQ111/Q111) expressing endogenous levels of normal and mutant huntingtin with 7 and 111 glutamines, respectively. We studied the protection of fetal and postnatal glia conditioned medium (GCM) on H2O2 (2 µM), glutamate (5 mM) and 3-nitropropionic acid (2.5 mM) related toxicity. We also compared the neuroprotective effects of GCM versus that of the growth factors bFGF, BDNF and GDNF. Fetal GCM protects from every toxin, reducing the cell death and increasing the cell survival. Fetal GCM reduces the caspases fragmentation of the protein PARP, the expression of chaperone Hsp70 and the accumulation of ROS and polyubiquitinated proteins. In addition, in Q111 striatal cells treated with H2O2 (2 µM) for 24 hours, the intracellular GSH levels are higher in the presence of GCM. Notably, the 13-day and 2-month postnatal GCM, totally protects from H2O2 induced cell death in mutant striatal cells. GCM neuroprotective effects are more potent than those of the already identified neurotrophic factors. We conclude that GCM protects Q111 cells from neuronal neurotoxins and the effects of GCM are more potent than those of any known neurotrophic factor. GCM may contain new and more potent, as yet unidentified, neurotrophic molecules, potentially useful in patients with Huntington’s disease.

## Introduction

Huntington’s disease (HD) is an autosomal dominant disorder characterized by progressive motor, cognitive and psychiatric disturbances 1, 2 associated to predominant neuronal loss in the striatum and other brain areas 3. HD is caused by an expanded chain of polyglutamines localized in the N-terminal region of the huntingtin protein^4^ . The physiological role of huntingtin and the pathogenic mechanisms that produce the disease are unknown. Mutant huntingtin produces fibrillary deposits; changes its normal localization, produces cytoplasmic and intranuclear inclusions; changes gene transcription; alters synaptic transmission; impairs mitochondrial activity and activates caspases and other pro-apoptotic molecules 5, 6, 7. The fact that the localization of huntingtin is ubiquitous, while there is a lesional gradient with more severe involvement of the striatum, suggests that some regional factors, such as synaptic connections and local metabolic rates play a role in neuronal degeneration.

Glutamate is considered to play a pathogenic role in HD mediated by metabotropic, and perhaps other, receptors 8 9. Reactive oxygen species (ROS) may play an important role in HD 10 and may be the initial molecules leading to apoptosis. Hydrogen peroxide (H_2_O_2_) is one of the major oxygen species which mediates cytotoxicity 11 , 12. Overproduction of ROS contributes to deregulation of transmitter release by increasing the extracellular level of glutamate and monoamine transmitters, which act as triggers of deleterious cellular events leading to neurodegeneration 13. In addition, mitochondria play a key role in the regulation of neuronal aging and activity of proteins related to neuronal death 14 , 15. 3-Nitropropionic acid (3-NP) is a natural toxin that irreversibly inhibits the succinate dehydrogenase enzyme, which is the main constituent of mitochondrial chain complex II. In rodents, 3-NP produces striatal lesions, mostly involving medium-sized spiny projection neurons; a pattern of lesions very similar to those of patients with HD 15.

Recent studies have revealed the role of glia in different neurodegenerative diseases, including HD 12, 16, 17, 18, 19, 20, 21. *In vitro*, glial cells release chemical substances into the media 22, 23, including antioxidants and neurotrophic factors. Glia-conditioned medium (GCM) is rich in antioxidants, namely: reduced glutathione (GSH) and ascorbic acid (AA); and peptidic growth factors: glial cell line–derived neurotrophic factor (GDNF), brain-derived neurotrophic factor (BDNF), nerve growth factor (NGF), and basic fibroblast growth factor (bFGF) as well as novel neurotrophic proteins 12, 24.

The aim of this study was to test the neuroprotective effect of GCM in a cellular neuronal model of HD. We chose the cell line STHdh^Q111/111^ (Q111), untreated or challenged with striatal neurotoxins, to model the molecular and cellular events that take place in HD. These cells are striatal progenitor cells from knock-in mouse models of HD, expressing expanded human huntingtin at baseline 25. These cells also reproduce several features observed in HD and in mouse models of this disease. Q111 cells exhibit impaired energy metabolism, reduced ATP and ATP/ADP ratio 25, 26, 27, as well as increased sensitivity to 3-NP 28, 29 and respiratory chain defects 30.

In this work, we have tested if fetal and postnatal mice GCM protected Q111 neurons from spontaneous cell death as well as from exposure to glutamate, oxidative damage and mitochondrial toxins.

## Material and methods

**Culture Media. **Dulbecco’s modified Eagle’s medium (DMEM) with high glucose (4.5 g/liter) was from Sigma (Madrid, Spain), Ham’s F12 nutrient mixture, Eagle’s minimal essential medium (EMEM) with Earl’s salts, Leibovitz’s L-15 medium; and supplements as L-glutamine, fetal bovine serum (FBS), sodium pyruvate, geneticin and L-glutamine, purchased from Invitrogen (Madrid, Spain). Glucose 45 %, trypsin-EDTA, insulin, putrescine, progesterone, and sodium selenite were from Sigma (Madrid, Spain), and human transferrin, 30 % iron saturated, was from Boehringer- Mannheim (Barcelona, Spain).

**Neuronal and glial cultures. **Conditionally immortalized, STHdh^Q7/Q7^ (Q7), and Q111 striatal neuronal progenitor cell lines expressing endogenous levels of huntingtin with 7 and 111 glutamines, respectively, were kindly provided by Prof. Alberch (Universitat de Barcelona, Spain) and Prof. Marcy E. Macdonald (Massachusetts General Hospital, Boston, USA) and were described previously by Trettel^ 25^ . Striatal cells were grown at 33 °C in Dulbecco’s Modified Eagle’s Medium (DMEM), supplemented with 10 % fetal bovine serum (FBS), 1 % streptomycin-penicillin, 2 mM L-glutamine, 1 mM sodium pyruvate, and 400 μg/ml geneticin (G418). Twenty-four hours after plating, the cells were changed to serum-free defined medium (EF12) as previously reported 31, 32. EF12 consisted of a 1:1 (v/v) EMEM and nutrient mixture of Ham’s F-12, supplemented with D-glucose (6 mg/ml), insulin (25 µg/ml), transferrin (100 µg/ml), putrescine (60 µM), progesterone (20 nM), and sodium selenite (30 nM).

Glial striatal cultures were obtained from 129SV/C57BL/6 wild-type (WT) mice as previously described 12. Glia-enriched striatal cultures were obtained from sibling cells kept in culture from 10–15 days to 3 months in DMEM-FBS. Positive staining with anti-GFAP antibody identified the astrocytes in these cultures. After 10–15 days in culture, the number of astrocytes was about 80–90 % of total cells 22. The cultures were fed once per week. To obtain the glia conditioned medium (GCM), DMEM-FBS medium was discarded, and the cells were washed out three times with Leibovitz’s L-15 and subsequently cultured in serum free defined medium. After 24 hours of culture under such conditions, the medium was collected and stored frozen. This medium was considered GCM.

**Antibodies. **The following antibodies were used: Anti-mouse IgG fluorescein from Jackson (PA, USA) and anti-rabbit IgG Alexa Fluor from Molecular Probes (Eugene, OR, USA). Mouse monoclonal anti-HSP-70 and rabbit polyclonal anti-CHIP antibodies were from Santa Cruz Biotechnology (Heidelberg, Germany). Mouse monoclonal anti-PARP was from Cell Signalling Technology (Denver, USA). Mouse monoclonal anti-ubiquitin, anti-synaptophysin and anti-ß-tubulin class III antibodies were from Chemicon, Millipore (Temecula, CA, USA). Anti-mouse and anti-rabbit horseradish peroxidase secondary antibodies were from Amersham. ß-Actin secondary antibody was an anti-mouse phosphatase alkaline conjugated from Sigma (Madrid, Spain).

**Chemicals. **5,5-Dithio-bis-2-nitrobenzoic acid (DTNB), hydrogen peroxide, sodium glutamate, 3-nitropropionic acid, trypan blue reagent and reduced glutathione (GSH) were from Sigma (Madrid, Spain). NADPH and GSH reductase (GR) were from Boehringer-Mannheim (Barcelona, Spain). The cytotoxicity detection kit for lactate dehydrogenase (LDH) and the cell proliferation kit I [3-(4,5-dimethylthiazol-2-yl)-2,5-diphenyl tetrazolium bromide (MTT)] were from Roche Diagnostics. The BCA protein assay kit was from Pierce (Rockford, Ill, USA). All other reagents were of the highest purity commercially available from Merck or Sigma.

**Treatment of the cells**Experiments were performed in 12 and 24-well culture plates for western blot and for survival and death assays, respectively. For the immunocytochemistry detection, the cells were cultured in 13 mm-diameter glass cover slips with a density of 5000 cells. After 3 days in vitro (DIV) in the growth medium (DMEM-FBS), Q7 and Q111 cells reached confluence. At this time, the medium was discarded and the cells were subsequently cultured and treated in a chemically serum-free defined medium (EF12) or in striatal glia-conditioned medium (GCM) (embryonic E16, postnatal of 13 days and postnatal of 2 months).

In order to test the protection of fetal GCM against different neurotoxins, we added the following chemicals to Q7 and Q111 cells cultured in defined medium, supplemented or not with fetal GCM: sodium glutamate (5 mM), H_2_O_2_ (2 µM), 3NP acid (2.5 mM) for 24 hours. In order to study the protection of postnatal GCM against H_2_O_2_ induced cell death in Q111 cells we used postnatal striatal GCM, from 13-day and 2-month old mice and the experiments were performed as described in the previous paragraph.

To investigate the mechanisms of neuroprotection by fetal GCM, we studied the ubiquitination of proteins, and the expression of death and chaperone proteins by Western blot assays. To compare the effects of GCM with the well-known neuronal growth factors, we treated Q111 cells with fetal GCM versus different growth factors such as GDNF (50 and 100 ng/ml), bFGF (10 and 20 ng/ml) and BDNF (50 and 100 ng/ml). We initially studied the effects of each growth factor on Q111 cells and compared them with the fetal GCM. Then, we studied the protection of the growth factors and fetal GCM on H_2_O_2_ induced cell death in Q111 cells.

**Cell survival, proliferation assay and cell differentiation. **Necrotic cell death was measured according to LDH activity in the culture medium and by trypan blue dye exclusion in cells. LDH activity was measured by using a cytotoxicity detection kit 12 , 33 . Apoptotic cell death was measured by nuclei stained with bis-benzimide (Hoechst 33342). Mitochondrial activity was analyzed with the 3-(4,5-dimethylthiazol-2-yl)-2,5-diphenyl tetrazolium bromide (MTT) assay in Q7 and Q111 cells. The MTT assay determines the ability of cells to metabolize MTT. At the end of the cell treatment period, 300 µl of culture medium were removed from total 500 µl of each well, and 20 µl of MTT solution (5 mg/ml) added and incubated for 2 h. At this time, 200 µl of solubilization solution (10 % SDS in 0.01 M HCl) were then added to the wells, and, after 24 h of incubation at 37 °C, 100 µl transferred into a 96-well microtiter plate. The absorption value at 540 nm was measured in an automatic microtiter reader (Spectra Fluor; Tecan, Männedorf, Switzerland). To test the differentiation of the cells, we counted the number of multiprocess cells as the number of cells with 3 or more prolongations.

**Immunocytochemistry. **After the experimental treatments, the cells were fixed with 4 % paraformaldehyde. Then, cells were postfixed and permeabilized in ethanol-acetic acid (19:1) for 10 min at -20 ºC and incubated in a blocking solution followed by overnight incubation at 4 ºC with the cytoplasmatic class III ß-tubulin primary antibody (1:300) and with a protein of the presynaptic vesicle exocytosis, synaptophysin primary antibody (1:200). Fluorescein and Alexa Fluor-conjugated secondary antibodies were used to visualize positive cells under fluorescent microscopy. Nuclei were stained by bis-benzimide (Hoechst 33342) and immunostaining was visualized under fluorescent microscopy. The number of immunoreactive cells was counted in predefined parallel strips.

**Measurement of GSH and ROS levels.**

Total glutathione levels were measured by the method of Tietze 34. Intracellular levels of ROS were detected using the non-fluorescence DCF dye, which is oxidized by intracellular ROS to form the highly fluorescence DCF. The cells were incubated with 5 µM DCFH-DA 30 min before H_2_O_2_ treatments. After washing the excess of DCF with a medium free of phenol red (PBS with 1mM glucose), the fluorescence was measured in a laser-scanning confocal microscope (Nikon eclipse Ti) or in an automatic microtiter reader (Spectra Fluor, Tecan) at excitation and emission of 485 nm and 582 nm, respectively. Fluorescence was quantified by automated image analysis with Image Pro software. For each section mean fluorescence was calculated from 7 separate single fields.

**Western blot analysis and detection of ubiquitinated proteins.**

Q111 extracts for western blot analysis were prepared in ice-cold extraction buffer consisting of 20 mM Tris–HCl (pH 7.4), 10 mM potassium acetate (AcK), 1 mM dithiothreitol (DTT), 0.25 % NP-40, 1 mM EDTA, 2 mM EGTA, 1 mM PMSF, protease inhibitors cocktail (SIGMA) and a cocktail of phosphatase inhibitors (100 mM sodium fluoride, 20 mM sodium molybdate and 20 mM ß-glicerophosphate). The samples were homogenized, centrifuged at 4 ºC and protein content was determined by the BCA protein assay kit. Total protein (30 µg) was electrophoresed in 10 % SDS-PAGE gels and transferred to 0.45 mm nitrocellulose membranes (Amersham), as described previously 12, 33. After blocking, blots were incubated overnight at 4 ºC in 5 % non- fat dried milk with primary antibodies: the chaperone anti-HSP-70 (1/700); anti-CHIP (1/1000) and anti-PARP (1/1000).

To determine changes in ubiquitination, cell cultures treated or untreated with H_2_O_2_ (2 µM) in serum-free defined medium and in GCM for 24 h, were scraped in 150 µl of lysis buffer [50mM Tris–HCl, 150 mM NaCl, 20 mM EDTA, 1 % TritonX-100, 50 mM sodium fluoride, 20 mM N-ethyl-maleimide, 100 µM sodium ortovanadate, 1 mM PMSF and protease inhibitors cocktail (SIGMA)] and boiled for 5 min. The lysates were centrifuged at 12 000 x g at 4 ºC for 30 min and 7.5 µg of protein were conducted to immunoblot assay with a rabbit polyclonal antibody to ubiquitin.

The secondary antibodies (1/2000) followed by ECL detection reagents (Amersham) were used for immunodetection. Immunoblot of ß-actin diluted (1/20000) was performed to demonstrate equal protein loading. The blots were quantified by computer-assisted video densitometry.

**Characterization of defined and glia-conditioned medium protein profile. **Defined E12 and embryonic E16 glia-conditioned media were lyophilized and concentrated in 5 % of initial volume. Protein concentration was determined by BCA method and 5 µg of protein from the supernatants of both media, were used for the electrophoresis in 4-20 % polyacrylamide gel. Electrophoresis was carried out at 150V. After completion of electrophoresis, the gel was developed using silver staining to observe protein profile.

**Statistical analysis. **The statistical analysis was performed by one-way analysis of variance (ANOVA), followed by Newman–Keuls multiple comparison test. Differences were considered statistically significant when p< 0.05. Analysis of data was performed using the Graph Pad Software (San Diego, CA) Prism 4 software.

## Results

**Fetal striatal GCM prevents cell death produced by sodium glutamate in Q7 and more so in Q111 cells. **Sodium glutamate, 5 mM x 24h, reduced cell proliferation and mitochondrial activity and increased cell death in Q7 and Q111 cells in serum-free defined medium (Fig 1A-C). After treatment with GCM the number of high affinity GABA uptake sites, a marker of striatal neurons, was reduced to around 75 % of controls (Fig 1D). Co-treatment, with fetal GCM completely reverted these effects of glutamate in Q111 cells (Fig 1A-D).

**Effects of fetal striatal glía-conditioned medium (GCM) on sodium glutamate induced toxicity in Q7 and Q111 cells:**
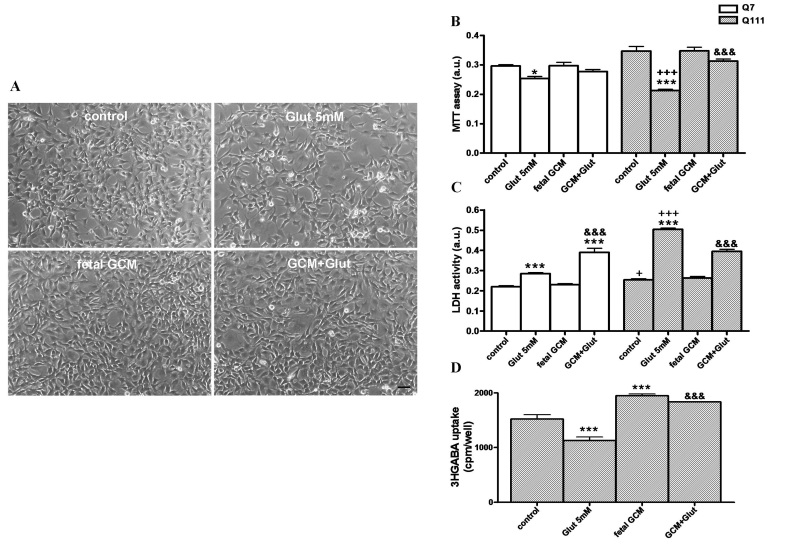
(A) Representative micrograph of Q111 cells after 3DIV in GCM or DM during 24 hours. Cells were treated with Glut 5 mM in DM or GCM (scale bar = 50 μm). (B) Mitochondrial activity (MTT assay). (C) Cell death measured by LDH assay. (D) 3H-GABA uptake in Q111 cells. The values are expressed as mean ± SEM (n = 5-6 samples in each experimental group). Statistical analysis was performed by one-way ANOVA followed by Newman-Keuls test. *p<0.05, ***p<0.001 vs control group. +p<0.05, +++p<0.001 Q111 cells vs Q7 cells. ^&&&^p<0.001 GCM + Glut vs Glut.

**The fetal striatal GCM prevents cell death produced by 3-nitropropionic acid in Q111 cells. **The mitochondrial complex II inhibitor (3NP, 2.5 mM x 24h) induced cell death in Q111 cells, as shown by reducing the neuronal area of the culture (Fig 2A and B) and increasing the number of apoptotic cells of around 50 % more than in the untreated cultures (Fig 2C and D). Co-treatment with fetal GCM increased the neuronal area of the cultures, expressed by the area of ß-tubulin (Fig 2A and B), and reduced the levels of apoptosis not only to levels of the cultures untreated with 3NP, but to levels below 50 % of those of the controls (Fig 2C and D).

**The glía-conditioned medium protects from 3-nitropropionic acid (3NP) toxicity in Q111 cells:**
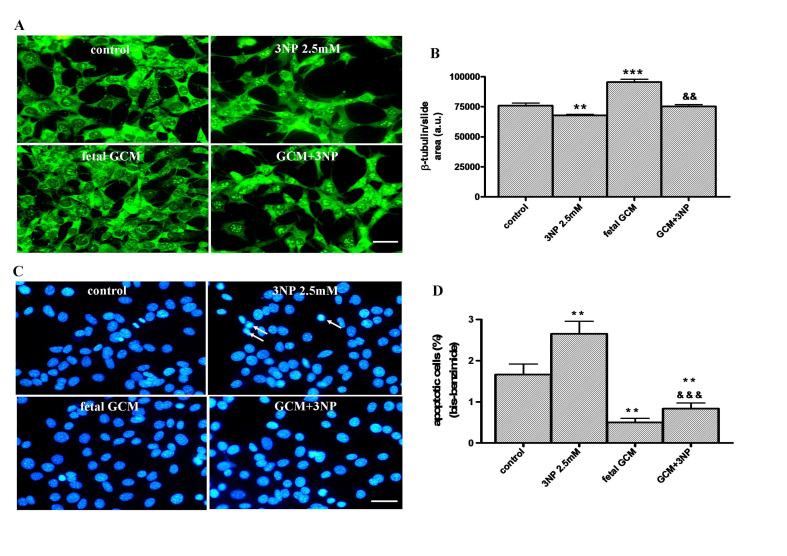
(A) Photomicrographs from control (DM), 3NP (2.5mM) and fetal striatal GCM treated cells for 24 h at 3 DIV (scale bar = 30 µm) and (B) their corresponding quantification of total neurons expressed as microtubule protein (β-tubulin). (C) Photomicrograph of total nuclei stained with bis-benzimide in the four experimental groups of Q111 cells. The arrows indicate the apoptotic cells in 3NP treated group. (D) Percentage of chromatin condensed and fragmented nuclei in the different experimental groups. The values are expressed as mean ± SEM (n = 4-6 samples in each experimental group). Statistical analysis was performed by one-way ANOVA followed by Newman-Keuls test. **p<0.01, ***p<0.001 vs control group. ^&&^p<0.01, ^&&&^p<0.001 GCM + 3NP vs 3NP.

**Fetal striatal GCM decreases H_2_O_2_ induced cell death in Q111 cells. **The treatment with H_2_O_2_, 2µM for 24 hours, decreased the ß-tubulin area of the cultures to around 2/3 of the controls (Fig 3A and B), almost doubled the percentage of apoptotic cells in the cultures (Fig 3C) and more than tripled the number of necrotic cells, as shown by trypan blue staining (Fig 3D and E). Co-treatment with fetal GCM completely prevented the toxic effects of H_2_O_2_, on ß-tubulin staining (Fig 3A and B) and apoptosis (Fig 3C) and reduced the percentage of necrosis to levels even below those of the controls (Fig 3D and E).

The treatment with H_2_O_2_, induced greater accumulation of intracellular ROS in Q111 cells than in Q7 cells (data not shown). The accumulation of ROS in Q111 cells was time (Fig 3F and G) and dose dependent (Fig 3H). Co-treatment with fetal GCM completely prevented the elevation of ROS induced by H_2_O_2_(Fig 3I and J).

**The glía-conditioned medium protects from cell death and intracellular ROS accumulation induced by H2O2 in Q111 cells:**
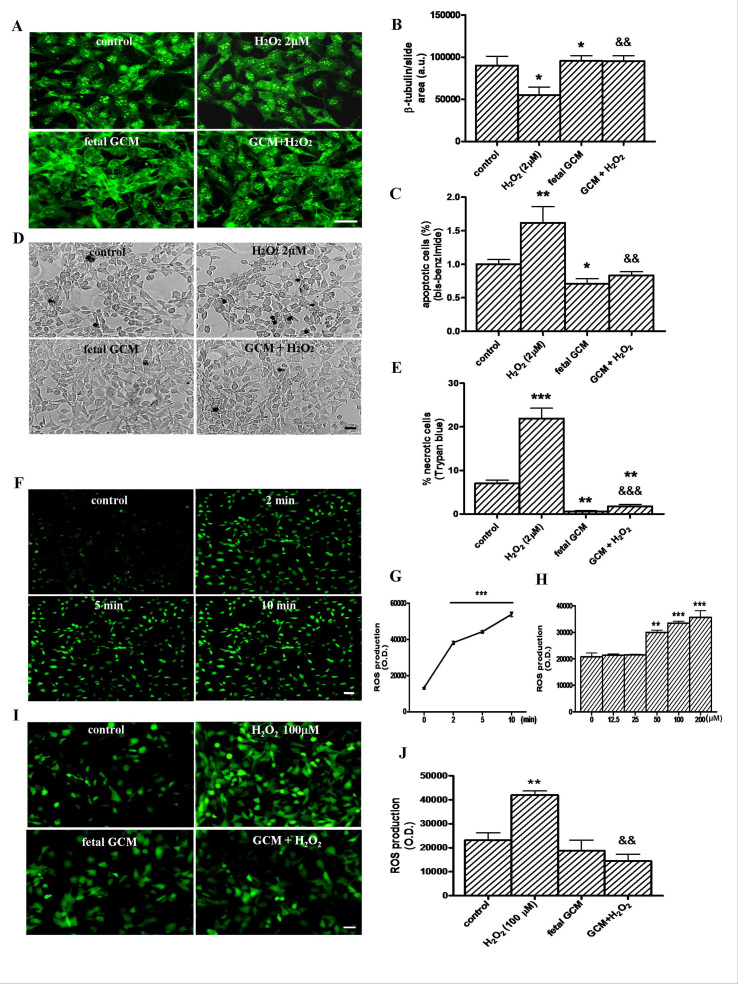
(A) Photomicrographs from control, H_2_O_2_ (2 µM) and fetal striatal GCM treated cells for 24 h at 3 DIV (scale bar = 30 µm) and (B) their corresponding quantification of total neurons expressed as microtubule protein (β-tubulin). (C) Percentage of chromatin condensed and fragmented nuclei in the different experimental groups. (D) Photomicrographs of cell death by trypan blue staining from the four experimental groups. (E) Percentage of necrotic cells stained with trypan blue in the four experimental groups. (F) Confocal photomicrographs of time-dependently ROS generation-induced by 100 μM H_2_O_2_ treatment (scale = 50 μm). (G) DCF fluorescence was measured with a reader at 485 nm of excitation and 582 nm emission and expressed in optical density units (O.D). (H) Quantification of dose-dependent ROS generation by H_2_O_2_ (12.5, 25, 50, 100 and 200 μM) for 10 min and expressed in O.D. (I) Photomicrographs with fluorescence microscope of pre-treatment with GCM 30 min before 100 μM H_2_O_2_ for 10 min prevented the ROS generation. (J) Quantification of intracellular ROS generation in the four experimental groups. The values are expressed as mean ± SEM (n = 4-6 samples in each experimental group). (Scale bar = 30 μm). Statistical analysis was performed by one-way ANOVA followed by Newman-Keuls test. *p<0.05, **p<0.01, ***p<0.001 vs control group. ^&&^p<0.01, ^&&&^p<0.001 GCM + H_2_O2 vs H_2_O_2_.

**Mechanisms implicated in the GCM neuroprotection in Q111 cells**

**Free radicals. **In order to study the mechanisms implicated in the GCM neuroprotection from H_2_O_2_ induced cell death, we investigated the differential pattern of proteins between defined medium (DM) and fetal GCM and the differences in the free radical scavengers. Gel electrophoresis of GCM and DM revealed the presence of seven bands in the GCM, which were absent or much weaker in DM. These bands have a relative molecular weight of 250, 123, 38, 36, 33, 29 and 12 KDa (Fig 4A). GSH levels were many folds greater in fetal GCM (0.58 ± 0.04) than in DM (0.013± 0.01). This may explain, at least in part, the GCM protective effects (Fig 4B). Treatment with H_2_O_2_, 2µM, decreased the intracellular levels of GSH and co-treatment with fetal GCM increased these levels even above the levels of the controls (Fig 4C).

**Apoptotic proteins. **The cleavage of PARP by caspases is recognized as a hallmark of apoptosis 35 . The H_2_O_2_ activated the accumulation of this cleavage and the fetal GCM prevented this effect. H_2_O_2_, 2µM x 24h, tripled the ratio PARP cleaved/full, an index of apoptosis, in Q111 cells and co-treatment with GCM greatly diminished this index (Fig 4D).

**Chaperones. **The chaperone HSP 70 levels were increased by treatment with H_2_O_2_, 2µM x 24h (Fig 4E) and these effects were prevented by fetal GCM (Fig 4E). The levels of CHIP were not changed by treatment with H_2_O_2_, 2µM x 24h, GCM or both (data not shown).

**Polyubiquitinated proteins**H_2_O_2_, 2µM x 24 hours, induced the accumulation of polyubiquitinated proteins in Q111 cells (Fig 4F and G). Co-treatment with GCM reverted this accumulation (Fig 4F and G).

**Mechanisms of GCM neuroprotection in Q111 cells:**
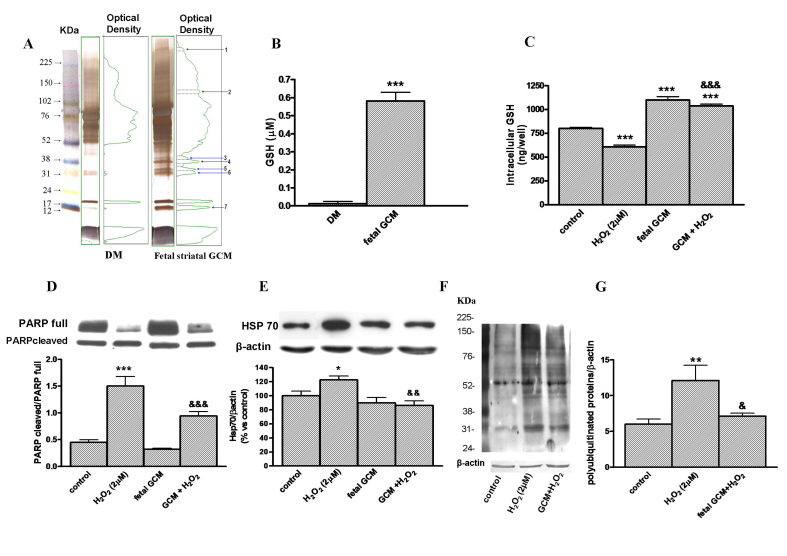
(A) Pattern of band proteins in GCM and DM. Gel electrophoresis of defined medium and fetal striatal glia-conditioned medium (GCM) and their corresponding densitometric scanning. Numbers 1 to 7 (250, 123, 38, 36, 33, 29 and 12 KDa respectively) on right panel indicate the seven bands only present in GCM. (B) Levels of glutathione in defined medium and fetal GCM. (C) Intracellular glutathione levels in Q111 cells treated with H_2_O_2_ (2 µM) and GCM for 24 h. (D) Expression of the full and cleaved, activated, forms of PARP in cells treated with H2O2 in DM or GCM. (E) Expression of chaperone Hsp70. (F) Accumulation of polyubiquitinated proteins induced by H_2_O_2_ in Q111 cells and (G) their corresponding densitometric analysis. The values are expressed as mean ± SEM (n = 4-6 samples in each experimental group). Statistical analysis was performed by one-way ANOVA followed by Newman-Keuls test. *p<0.05, **p<0.01, ***p<0.001 vs control group. ^&^p<0.05, ^&&^p<0.01, ^&&&^p<0.001 GCM + H_2_O_2_ vs H_2_O_2_.

**Postnatal GCM prevents cell death produced by H_2_O_2_ in Q111 cells. **We have also studied the protective effects of postnatal GCM, of 13 days and 2 months in the huntingtin mutant cells (Fig 5). Postnatal GCM prevents H_2_O_2_ induced cell death in Q111 cells. GCM prevented the H_2_O_2_-induced reduction of the ß-tubulin area and the increased apoptosis in Q111 cells (F 5A-H).

**The 13-day and 2-month postnatal striatal glial conditioned medium protects from H2O2 toxicity in Q111 cells:**
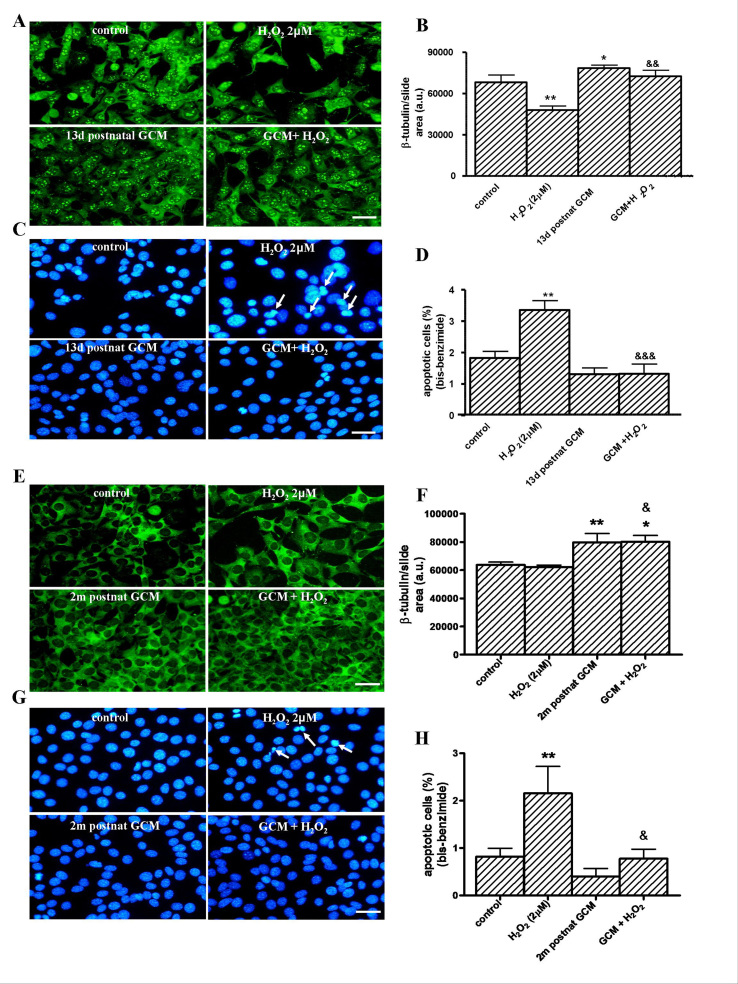
(A) Photomicrographs of control, H_2_O_2_ (2 µM) and 13 day postnatal striatal GCM treated cells for 24 h at 3 DIV (scale bar = 30 µm) and (B) their corresponding quantification of total neurons expressed as microtubule protein (ß-tubulin). (C) Photomicrograph of total nuclei stained with bis-benzimide in the four experimental groups of Q111 cells. The arrows indicate the apoptotic cells produced by the H_2_O_2_ toxicity. (D) Percentage of chromatin condensed and fragmented nuclei in the different experimental groups. (E) Photomicrographs of control, H_2_O_2_ (2 µM) and 2 month postnatal striatal GCM treated cells for 24 h at 3 DIV (scale bar = 30 μm) and (F) their corresponding quantification of total neurons expressed as microtubule protein ß-tubulin. (G) Photomicrographs of total nuclei stained with bis-benzimide in the four experimental groups of Q111 cells. The arrows indicate the apoptotic cells produced by the H_2_O_2_ toxicity (H) Percentage of chromatin-condensed and fragmented nuclei in the different experimental groups. The values are expressed as mean ± SEM (n = 4-6 samples in each experimental group). Statistical analysis was performed by one-way ANOVA followed by Newman-Keuls test. *p<0.05, **p<0.01 vs control group. ^&^p<0.05, ^&&^p<0.01, ^&&&^p<0.001 GCM + H_2_O_2_ vs H_2_O_2_.

**Comparative effects of neurotrophic factors and GCM on Q111 cells**

**The growth factor bFGF has beneficial effects in Q111 cells but fewer than fetal GCM. **Basic FGF 10 and 20 ng/ml, did not change the neuronal area of the cultures, as measured by the ß-tubulin staining, a parameter which is increased by GCM (Fig 6 A and B). Both doses of bFGF decreased the percentage of apoptotic cells (Fig 6 C and D) and increased the cell differentiation measured by the number of cells with multiprocesses (Fig 6 E and F), but the effects of bFGF, at the doses used in these experiments, were less potent than those of GCM.

**The effects of growth factor bFGF and the fetal striatal glia-conditioned in Q111 cells:**
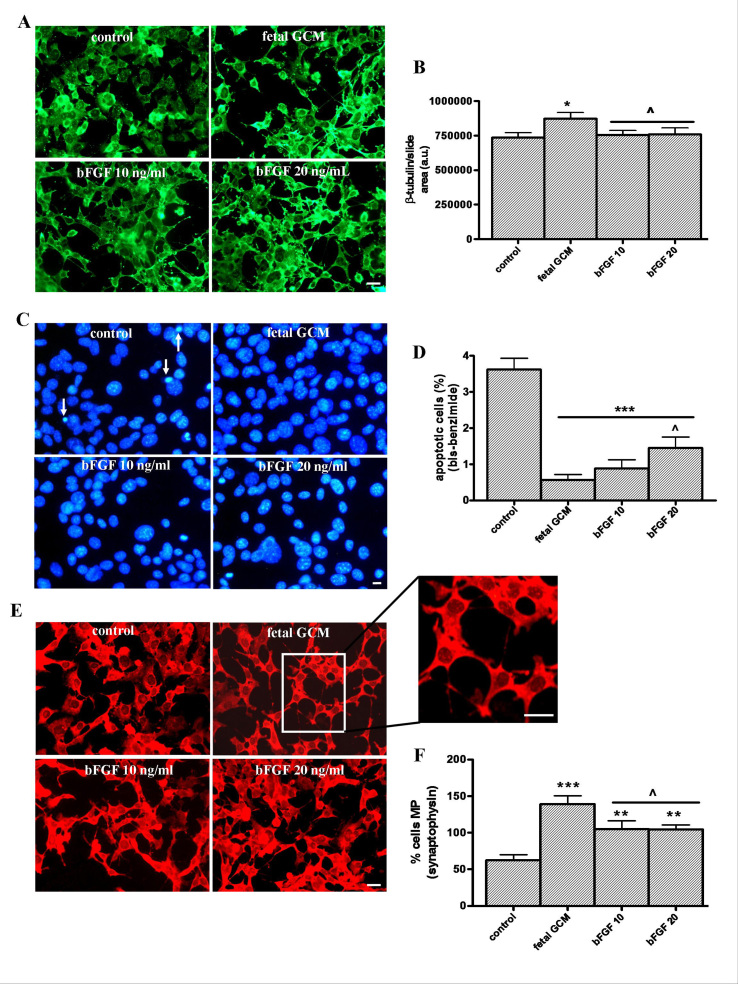
(A) Photomicrographs of control, fetal striatal GCM, bFGF (10 ng/mL) and bFGF (20 ng/mL) treated cells for 24 h at 3 DIV (scale bar = 30 µm) and (B) their corresponding quantification of total neurons expressed as microtubule protein (ß-tubulin). (C) Photomicrographs of total nuclei stained with bis-benzimide in the four experimental groups of Q111 cells (scale bar = 30 µm). The arrows indicate the apoptotic cells. (D) Percentage of chromatin condensed and fragmented nuclei in the different experimental groups. (E) Photomicrographs of synaptophysin staining (scale bar = 30 µm). A portion of this micrograph has been magnified in the picture on the right. (F) Percentage of cells with multiprocesses, cells with more than 3 neurite arborization, to test the cell differentiation. The values are expressed as mean ± SEM (n = 6 samples in each experimental group). Statistical analysis was performed by one-way ANOVA followed by Newman-Keuls test. *p<0.05, **p<0.01, ***p<0.001 vs control group. ^p<0.05 bFGF vs fetal striatal GCM.

**The growth factor GDNF has beneficial effects in Q111 cells but fewer than fetal GCM. **GDNF, 50 and 100 ng/ml, did not increase, as GCM does, the ß-tubulin area of the cultures (Fig 7A and B) and had a significant but very mild protective effect on apoptosis, which is strongly suppressed by GCM (Fig 7C and D). GDNF 50 ng/ml failed to increase the percentage of cells with multiple processes, GDNF, 100ng/ml increased it by around 25 %; however, GCM almost doubled it (Fig 7E and F). As a consequence, GCM was a much more potent neurotrophic agent for Q111 cells than GDNF at doses used in this study.

**The growth factor GDNF and the fetal striatal glia-conditioned medium protect from H2O2 induced cell death in Q111 cells:**
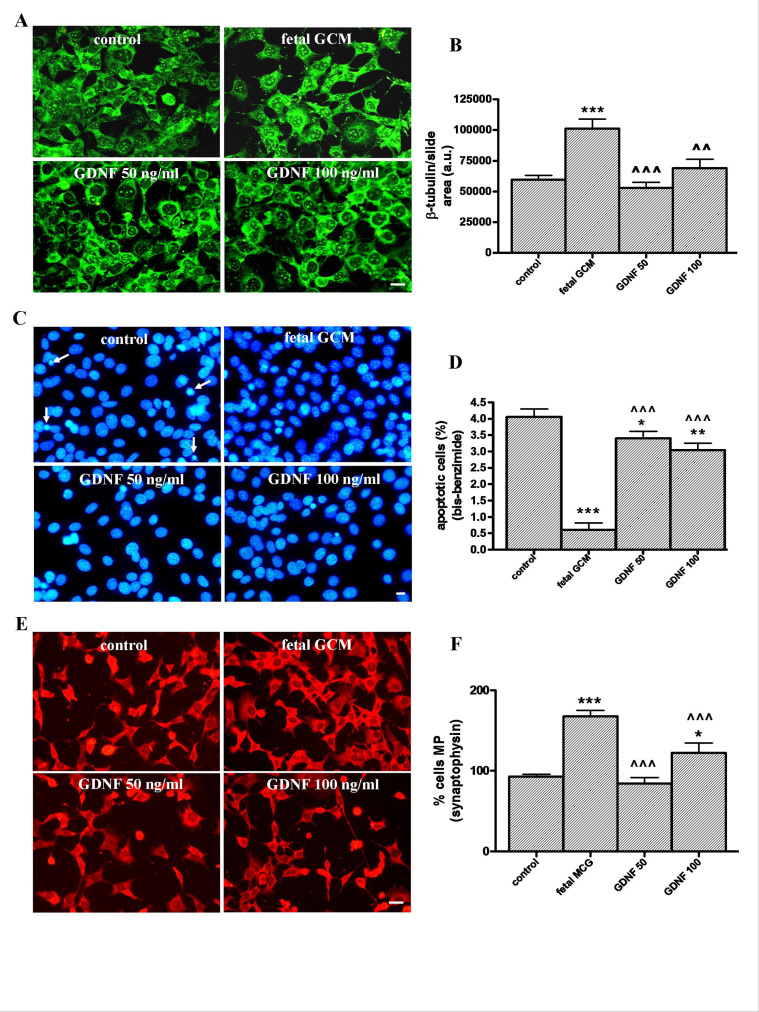
(A) Photomicrographs of control, fetal striatal GCM, GDNF (50 ng/mL) and GDNF (100 ng/mL) treated cells for 24 h at 3 DIV (scale bar = 10 µm) and (B) their corresponding quantification of total neurons expressed as microtubule protein (ß-tubulin). (C) Photomicrographs of total nuclei stained with bis-benzimide in the four experimental groups of Q111 cells (scale bar = 30 µm). The arrows indicate the apoptotic cells. (D) Percentage of chromatin condensed and fragmented nuclei in the different experimental groups. (E) Photomicrographs of synaptophysin staining (scale bar = 30 µm). (F) Percentage of cells multiprocesses, cells with more than 3 neurite arborization, to check the cell differentiation. The values are expressed as mean ± SEM (n = 6 samples in each experimental group). Statistical analysis was performed by one-way ANOVA followed by Newman-Keuls test. *p<0.05, **p<0.01, ***p<0.001 vs control group. ^^p<0.01, ^^^p<0.001 GDNF vs fetal striatal GCM.

**Effects of growth factor BDNF and fetal GCM in Q111 cells. **BDNF, 50 and 100 ng/ml, did not increase, as GCM does, the ß-tubulin area of the cultures (Fig 8A and B) and had a significant but smaller protective effect on apoptosis than GCM (Fig 8C and D). BDNF, 50 ng/ml, failed to increase the number of differentiated cells with multiple processes. BDNF, 100 ng/ml, increased this number significantly, with respect to control, but was less potent than GCM (Fig 8E and F). As a consequence, GCM was a much more potent neurotrophic agent for Q111 cells than BDNF at doses used in this study.

**The growth factor BDNF and the fetal striatal glia-conditioned medium protect from H2O2 induced cell death in Q111 cells:**
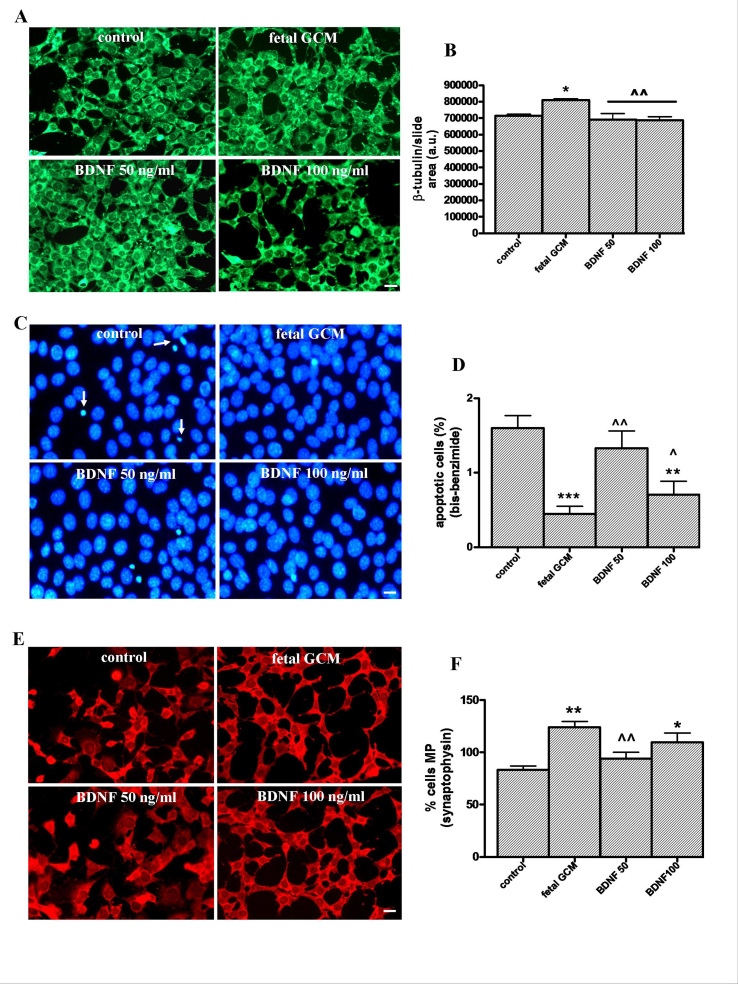
(A) Photomicrographs of control, fetal striatal GCM, BDNF (50 ng/ml) and BDNF (100 ng/ml) treated cells for 24 h at 3 DIV (scale bar = 10 µm) and (B) their corresponding quantification of total neurons expressed as microtubule protein (ß-tubulin). (C) Photomicrograph of total nuclei stained with bis-benzimide in the four experimental groups of Q111 cells (scale bar = 30 µm). The arrows indicate the apoptotic cells. (D) Percentage of chromatin-condensed and fragmented nuclei in the different experimental groups. (E) Photomicrograph of synaptophysin staining (scale bar = 30 µm). (F) Percentage of multiprocess cells, (with more than 3 neurite arborization) to test the cell differentiation. The values are expressed as mean ± SEM (n = 6 samples in each experimental group). Statistical analysis was performed by one-way ANOVA followed by Newman-Keuls test. *p<0.05, **p<0.01, ***p<0.001 vs control group. ^p<0.05, ^^p<0.01, ^^^p<0.001 BDNF vs fetal striatal GCM.

**Comparative effects of growth factors and fetal striatal GCM on H_2_O_2_ induced cell death. **We compared the effects of bFGF, GDNF, BDNF and fetal GCM on H_2_O_2_ induced toxicity. BDNF and bFGF failed to revert the H_2_O_2_ induced reduction of the ß-tubulin area of the cultures while GDNF and GCM did (Fig 9A and B). BDNF, GDNF and GCM prevented the H_2_O_2_ induced apoptosis, being GCM the most effective (Fig 9C) while bFGF failed to decrease apoptosis. None of the neurotrophic factors, at the doses used in this study, reverted the reduction of differentiated cells induced by H_2_O_2_, but GCM completely normalized it (Fig 9D and E). As a consequence, GCM was a more potent neurotrophic agent for Q111 cells than any of the neurotrophic factors at the doses used in this study.

**Comparison of bFGF, BDNF, GDNF and the fetal striatal GCM, neuroprotection from H2O2 induced cell death in Q111 cells:**
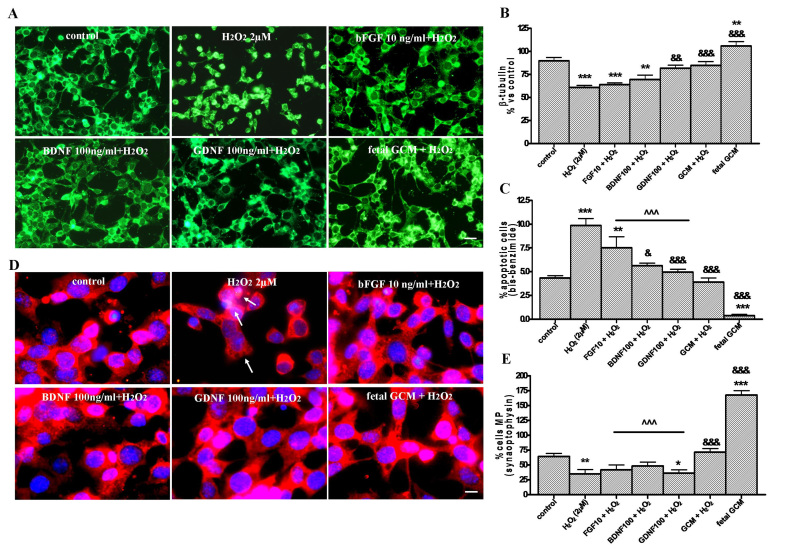
(A) Photomicrographs of control, H_2_O_2_ (2 µM), bFGF 10 ng/ml + H_2_O_2_, BDNF 50 ng/ml + H_2_O_2_, GDNF 50 ng/ml + H_2_O_2_, GCM + H_2_O_2_ treated cells for 24 h at 3 DIV (scale bar = 30 µm) and (B) their corresponding quantification of total neurons expressed as microtubule protein (ß-tubulin). (C) Percentage of chromatin condensed and fragmented nuclei in the different experimental groups. (D) Photomicrograph of synaptophysin staining (red) and Hoechst staining (blue) (scale bar = 15 µm). (E) Percentage of multiprocess cells, (with more than 3 neurite arborization) to test the cell differentiation. The values are expressed as mean ± SEM (n = 4 samples in each experimental group). Statistical analysis was performed by one-way ANOVA followed by Newman-Keuls test. *p<0.05, **p<0.01, ***p<0.001 vs control group. ^&^p<0.05, ^&&^p<0.01, ^&&&^p<0.001 growth factor or GCM + H_2_O_2_ vs H_2_O_2_,; ^^^p<0.001 growth factor + H_2_O_2_ vs fetal striatal GCM + H_2_O_2_.

## Discussion

These studies reveal that fetal and postnatal striatal GCM protects from spontaneous degeneration and cell death induced by exposure to glutamate, 3-NP acid and H_2_O_2_ in striatal cells with polyglutamines expansions of huntingtin which could be considered as an in vitro models of HD. GCM is rich in small antioxidants, like GSH and ascorbic acid, and peptidic neurotrophic factors, such as GDNF, BDNF, neurotrophin-3 (NT-3), NGF, and bFGF as well as novel DA neurotrophic factors 22, 36, 37, 38, 39, 40, 41, 42, 43. The effects of GCM observed in these experiments are, however, more potent than those of any known neuroprotective or neurotrophic factor ever tested in vitro models of HD.

All the mechanisms of neurotoxicity triggered by the neurotoxins used in these experiments, including abnormal excitatory neurotransmission, abnormal mitochondrial function, and excessive oxidative stress have been demonstrated in patients with HD and animal models of this disease 44, 45, 46. Glutamate is a major excitatory amino acid neurotransmitter in the central nervous system. A high concentration of glutamate induces neuronal cell damage under in vitro conditions 47, 48, 49. Striatal medium spiny neurons, which receive a strong excitatory input in their dendritic spines from cortical neurons, through the cortico striatal pathways, are neurons of projection mostly to the pallidum, which contain GABA and other neurotransmitters, and are particularly vulnerable to HD. Their progressive depletion is in strong correlation with symptom severity 50. Our data shows that the GCM reverted cell death produced by glutamate and increased the levels of GABA uptake sites, a marker of GABA neurons, in STHdhQ111/111 cells.

In HD there is a mitochondrial dysfunction that produces a significant decrease in the activity of mitochondrial respiratory chain complexes (MCC) II, III and IV, among other factors^ 51^. 3-Nitropropionic acid is a natural toxin that irreversibly inhibits the succinate dehydrogenase enzyme, which is the main constituent of MCC II. Administered to rodents, 3-NP reproduces the brain lesions observed in HD patients, which consist of degeneration of the striatum with particular impact on medium-sized spiny projection neurons 52. In Q111 cells, we have observed that the 3-NP produces a decrease in cell survival and an increase in necrotic and apoptotic cell death. This study shows the protection by GCM of 3-NP induced cell death.

We challenged Q111 cells with H_2_O_2_ to study the effects of the fetal and postnatal GCM on free radical metabolism and ubiquitinated proteins in Q111 cells. H_2_O_2_ produces an accumulation of free radicals and polyubiquitinated proteins in Q111 cells and fetal GCM reverts both nearly to control levels. Fetal GCM also prevents the H_2_O_2_ induced cell death in Q111 cells. The cleavage of PARP by caspases is recognized as a hallmark of apoptosis 35. H_2_O_2_ activated the accumulation of this cleavage and fetal GCM prevented this effect. The chaperone HSP70 is increased in the presence of H_2_O_2_ as a marker of cell stress but the fetal GCM reduced it. Fetal GCM increased the levels of the antioxidant GSH in Q111 cells and prevented the H_2_O_2_ induced depletion of GSH. The 13-day and 2-month postnatal GCM also decreased the H_2_O_2_ induced cell death in this in vitro model of HD.

Since GCM is rich in neurotrophic factors we investigated whether the effects of GCM could be related to the presence of these factors. We compared the effects of GCM and bFGF, GDNF and BDNF, at different concentrations, on one of the models of toxicity for Q111 cells used in this study; the challenge with H_2_O_2_. We found that GCM was more potent than any of the above mentioned factors for the rescue of Q111 cells, the reduction of apoptosis and the induction of arborisation, after treatment with H_2_O_2_. However, glia-expressing mutant huntingtin can also secret toxic molecules 53 54 55 56

We conclude that the GCM is a powerful neuroprotective agent for Q111 striatal cells. GCM should be tested on animal models of HD and, if the results obtained in this study are confirmed, on patients with HD.
